# Books and Magazines

**Published:** 1905-08

**Authors:** 


### Books and Magazines.

HUMAN PHYSIOLOGY. Prepared with Special Reference to Students of Medicine. By Joseph H. Raymond, A. M. A., M. D., Professor of Physiology and Hygiene, Long Island College Hospital, New York City. Third edition, thoroughly revised. Octavo volume of 687 pages, containing 444 illustrations, some in colors, and four full-page lithographic plates, Philadelphia and London. W. B. Saunders & Company, 1905. Cloth, $3.50 net.

It is evident that in revising his excellent work for the new third edition, Dr. Raymond spared no labor to bring it up to present-day knowledge. Every page shows evidence of his careful revision, and in that portion devoted to the physiology of nutrition the author has profitably availed himself of Chittenden's valuable contributions to the subject. Besides more fully elaborating many topics previously discussed in the old edition, the author has introduced a number of new subjects, among which are the Influences of Alcoholic Fluids on the Excretion of Uric Acid, Hemolysis and Bacteriolysis, and Ovarian and Abdominal Pregnancy. Dr. Raymond seems in some way to know just what features to illustrate and just what kind of illustrations to use, for every one of the 444 figures is practical and illustrates a point which might not be clear to the student. In would be difficult to find another volume of the same size containing so much up-to-date and accurate information.

---

A Text-Book of the Practice of Medicine. — For Students and Practitioners. By Hobart Amory Hare, M. D., B. Sc., Professor of Therapeutics and Materia Medica in the Jefferson Medical College of Philadelphia; Physician to the Jefferson Medical College Hospital; Laureate of the Royal Academy of Medicine in Belgium and of the Medical Society of London. Author of "A Text-Book of Practical Therapeutics; A Text-Book of Practical Diagnosis," etc. In one very handsome octavo volume of 1120 pages, with 129 engravings and 10 full-page plates in colors and monochrome. Cloth, $5, net; leather, $6, net; half Morocco, $6.50, net. Lea Brothers & Co., Philadelphia and New York, 1905.

This volume embodies the experience of Professor Hare of more than twenty years of actual hospital and private practice, during which time he has been constantly teaching the subjects of clinical medicine and therapeutics. All physicians are familiar with his splendid works on therapeutics, and they know the value and accuracy of his observations and his painstaking record of them. This work should, and no doubt will, meet with an active and steady demand.

---

The Treatment of Fractures, with Notes on a Few Common Dislocations. By Charles L. Scudder, M. D., Surgeon to the Massachusetts General Hospital. Fifth edition revised and enlarged. Octavo of 563 pages, with 739 illustrations. Philadelphia and London. W. B. Saunders & Company, 1905. Polished Buckrum, $5.00 net; Half Morocco, $6.00 net.

Every year for the past five years it has been our pleasure and profit to review a new edition of this excellent work by Dr. Scudder. It is, indeed, a most remarkable book, and the author and publishers are to be congratulated upon its publication. In this, the fifth edition, Dr. Scudder has added some fifty new illustrations, many of them X-ray plates, illustrating the actual line of fracture. The text also has been very carefully revised, and new matter added throughout. Important changes have been made in the treatment of fractures of the neck of the femur, bringing this part of the book in accord with the latest advances. The 739 illustrations do what they should—they illustrate, showing the reader just what is intended. Undoubtedly this fracture has aided greatly in the success of Dr. Scudder's work.

---

THE SURGICAL ASSISTANT, A MANUAL FOR STUDENTS, PRACTITIONERS, HOSPITAL INTERNES AND NURSES. By Walter M. Brickner, B. S., M. D., Assistant Surgeon, Mt. Sinai Hospital, Out-Patient Department, etc., 360 pages, 123 original illustrations and 116 illustrations of surgical instruments. New York. The International Journal of Surgery Co., 1905. Price, $2 net.

This splendid manual is one of the really important books of the year, inasmuch as it fills a place in medical literature that has hitherto been unfilled. It certainly meets a widespread demand in a highly acceptable manner, and it is sure to attain immediate and lasting popularity.

The book is not too large for the doctor's overcoat pocket, nor the nurse's satchel, yet it covers the entire subject. It is a book that no young practitioner should be without, as it will prove of the greatest value to him in his everyday work. Likewise it should be in the hands of every nurse and hospital interne. Its use will not only assure greater efficiency in everything pertaining to surgical operations, but will prevent all the embarrassing delays and annoyances caused by inexperience or lack of knowledge.

---

DISEASES OF THE KIDNEY, DISEASES OF THE SPLEEN, AND HEMORRHAGIC DISEASES. By Drs. H. Sentor and M. Litten, of Berlin. Edited, with additions, by James B. Herrick, M. D., Professor of Medicine in Rush Medical College, Chicago. Octavo of 816 pages, illustrated. Philadelphia and London. W. B. Saunders & Company, 1905. Cloth, $5.00 net; Half Morocco, $6.00 net.

With the appearance of this, the eleventh volume of Saunders' American edition of Nothnagel's Practice, the work nears completion, the final volume on the Heart being now in active preparation. Like the others, this volume can be taken as the acme of knowledge on the subjects embraced. Professor Senator's clear style, systematic arrangement of facts, and logical reasoning make his articles on the kidney indispensible to the practitioner. The editor, Dr. Herrick, has enlarged on certain points whenever necessary, especially regarding treatment, diagnosis, urinary analysis, etc., so as to increase the value of the work to the general practitioner. He has also added articles on Cryoscopy and Phloridzin Glycosuria.

The sections on the Spleen and the Hemorrhagic Diseases were written by Professor Litten, whose pioneer work in these fields is widely known. The articles on the Mosquito and its relation to Malaria, on Splenic Anemia, on Congenital Icterus with Splenomegaly, and on the X-rays in the treatment of Leukemia have been brought down to date by the editor. Indeed, the editor's interpolations add greatly to the practical value of the volume, and we are sure such an authoritative work on these subjects has never before been published.

---

THE DOCTOR'S WINDOW. Vol. V of *The Doctor's Recreation Series*, Charles Wells Moulton, General Editor. By Ina Russelle Warren. With an introduction by William Pepper, M. D., LL. D., 1904. The Saalfield Publishing Co., Chicago, Akron, O., New York. 8vo. Pp. 288. Gilt top. Cloth, $2.50; half Morocco, $4.

When the doctor gets tired reading about "interesting cases" and about "pus" and "bile" and about what is "good for" certain cases, like a horse at the end of winter—burnt out on "dry forage"—he turns naturally to pastures new and fields that are green. He wants a change of diet. We recommend this book as an appetizer. It is a poetical kaleidoscope. It contains all sorts of poems of, for, about and by doctors, "from grave to gay, from lively to serene." It is wholesome reading and will promote digestion.

